# Effects of Atrazine exposure on human bone marrow-derived mesenchymal stromal cells assessed by combinatorial assay matrix

**DOI:** 10.3389/fimmu.2023.1214098

**Published:** 2023-07-31

**Authors:** Crystal C. Uwazie, Bonnie M. Pirlot, Tyler U. Faircloth, Mihir Patel, Rhett N. Parr, Halie M. Zastre, Peiman Hematti, Guido Moll, Devi Rajan, Raghavan Chinnadurai

**Affiliations:** ^1^ Department of Biomedical Sciences, Mercer University School of Medicine, Savannah, GA, United States; ^2^ Department of Medicine, University of Wisconsin Madison, School of Medicine and Public Health, University of Wisconsin-Madison, Madison, WI, United States; ^3^ BIH Center for Regenerative Therapies (BCRT) and Berlin-Brandenburg School for Regenerative Therapies (BSRT), Charité Universitätsmedizin Berlin, Corporate Member of Freie Universität Berlin and Humboldt Universität zu Berlin, and Berlin Institute of Health (BIH), Berlin, Germany; ^4^ Department of Nephrology and Internal Intensive Care Medicine, Charité Universitätsmedizin Berlin, Corporate Member of Freie Universität Berlin and Humboldt Universität zu Berlin, and Berlin Institute of Health (BIH), Berlin, Germany

**Keywords:** mesenchymal stromal/stem cells (MSCs), combinatorial assay matrix technology, immunomodulation and regeneration, cellular phenotype and function, environmental herbicide atrazine

## Abstract

**Introduction:**

Mesenchymal Stromal/Stem cells (MSCs) are an essential component of the regenerative and immunoregulatory stem cell compartment of the human body and thus of major importance in human physiology. The MSCs elicit their beneficial properties through a multitude of complementary mechanisms, which makes it challenging to assess their phenotype and function in environmental toxicity screening. We here employed the novel combinatorial assays matrix approach/technology to profile the MSC response to the herbicide Atrazine, which is a common environmental xenobiotic, that is in widespread agricultural use in the US and other countries, but banned in the EU. Our here presented approach is representative for screening the impact of environmental xenobiotics and toxins on MSCs as an essential representative component of human physiology and well-being.

**Methods:**

We here employed the combinatorial assay matrix approach, including a panel of well standardized assays, such as flow cytometry, multiplex secretome analysis, and metabolic assays, to define the phenotype and functionality of human-donor-derived primary MSCs exposed to the representative xenobiotic Atrazine. This assay matrix approach is now also endorsed for characterization of cell therapies by leading regulatory agencies, such as FDA and EMA.

**Results:**

Our results show that the exposure to Atrazine modulates the metabolic activity, size, and granularity of MSCs in a dose and time dependent manner. Intriguingly, Atrazine exposure leads to a broad modulation of the MSCs secretome (both upregulation and downmodulation of certain factors) with the identification of Interleukin-8 as the topmost upregulated representative secretory molecule. Interestingly, Atrazine attenuates IFNγ-induced upregulation of MHC-class-II, but not MHC-class-I, and early phosphorylation signals on MSCs. Furthermore, Atrazine exposure attenuates IFNγ responsive secretome of MSCs. Mechanistic knockdown analysis identified that the Atrazine-induced effector molecule Interleukin-8 affects only certain but not all the related angiogenic secretome of MSCs.

**Discussion:**

The here described Combinatorial Assay Matrix Technology identified that Atrazine affects both the innate/resting and cytokine-induced/stimulated assay matrix functionality of human MSCs, as identified through the modulation of selective, but not all effector molecules, thus vouching for the great usefulness of this approach to study the impact of xenobiotics on this important human cellular subset involved in the regenerative healing responses in humans.

## Graphical Abstract

Profiling MSC Response to the Environmental Xenobiotic Herbicide Atrazine with Combinatorial Assay Matrix Technology. A panel of well-standardized profiling assays was employed to study the dose-and-time-dependent response of resting and IFNγ cytokine-stimulated/activated MSCs, thereby identifying the release of the chemokine IL-8 as a key marker for MSC responsiveness to Atrazine.

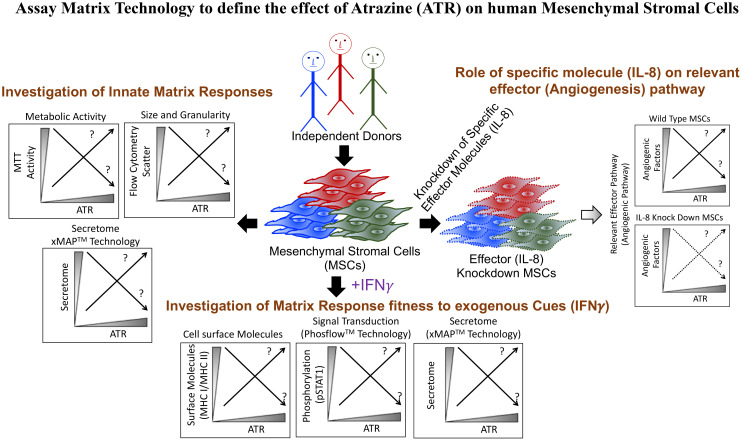

## Introduction

Environmental xenobiotics are currently mainly tested in animal models and cell lines to identify the effect of their exposure on visceral organ toxicity and systemic injury in the mammalian system (1–3). Although this strategy provides valuable insights on the systemic health effects of xenobiotics, the outcome of xenobiotic exposure on relevant human cellular targets, e.g. the regenerative stem cell compartment, still need to be defined better. Unlike terminally differentiated somatic cells, Mesenchymal Stromal/Stem Cells (MSCs) are the highly plastic and adaptive immunomodulatory non-hematopoietic stem cells of the human body that perform crucial functions in immune and tissue homeostasis in response to injury and inflammation (4–6). MSCs originate from the progenitors of the mesenchymal layer during embryonic development and can be identified in several tissues, such as bone marrow, adipose, placenta, umbilical cord etc. (7–11). Recently, key efforts have been undertaken to better define MSC phenotype and function (11–14). Profiling the effect of xenobiotic exposure on MSCs can identify modulated or non-modulated molecular pathways and properties associated with the functionality of these cells. These may provide key insights on the long-term health effects related to immune homeostasis imbalance, inflammation and injury in humans.

To determine the effect of xenobiotics on MSCs, suitable surrogate outcome measures of cell functionality need to identified and defined (11, 14). An appropriate understanding of MSC’s *in vivo* mechanism of action (MoA) in executing homeostasis, regenerative, and immunomodulatory functions may be beneficial in this aspect (15–19). The definition of effector molecules/pathways in executing MSC functions is still ongoing (19–26). The variability of individual effector functions of MSCs may depend on host genetics and other predisposing factors, which makes it difficult to identify single universal surrogate measures of functionality (11, 25–29). Thus, investigating a single well-defined MSC characteristic may be useful in some cases (11, 14), but not be appropriate as a broad surrogate measure of functionality in others. Alternatively, a combination of analytical assays that capture multiple properties associated with MSC phenotype and function may be used, here coined: “Combinatorial Assay Matrix Technology” (30–33). Here, a panel of well-defined and standardized assays, e.g. flow cytometry analysis of surface markers, secretome analysis of bioactive molecules, and phosphorylation status of MSCs, are collectively investigated as surrogate measures of MSC’s functionality. The combinatorial assay matrix technology may effectively identify MSC effector pathways and molecules modulated by xenobiotics exposure.

The xenobiotic Atrazine (C8H14ClN5), a chlorotriazine herbicide, is an herbicide used to prevent weed emergence in the United States, while banned in the European Union (34). Several studies have confirmed that Atrazine is the most common contaminant in the surface and drinking waters, and in the natural water reservoirs of the United States (35–39). Atrazine’s health implications have been widely discussed, and previous bodies of work have identified the side effects of Atrazine exposure on fresh water vertebrates (40–42). In addition, animal model studies have also determined the adverse effects of Atrazine exposure on neonatal development, immune function, central nervous system and endocrine system (43–49).

Previous studies demonstrated that pesticides at large can have an impact on MSC biology (50, 51). However, the effect of Atrazine on MSCs is largely unknown. Due to Atrazine’s substantial environmental significance and the principle lack of knowledge of its effect on MSC biology, we deployed assay matrix technology to define the effect of Atrazine on the multicomponent functionality of human MSCs (**Graphical Abstract**). Considering the host variability of MSC functions, our analysis included MSCs derived from the bone marrow of at least four to eight independent donors. We utilized a combinatorial analytical system including multiplex secretome analysis, metabolic assays, and flow cytometry. MSCs’ innate fitness was first defined with metabolic assays and secretome analysis focusing on immunomodulatory and regenerative factors upon exposure to Atrazine. Alternatively, the effect of Atrazine exposure on MSC’s responsive fitness was evaluated by analyzing IFNγ-induced phosphorylation, immunomodulation of surface molecules and secretome using Phosflow, flow cytometry and multiplex secretome analysis. This assay matrix technology has cumulatively determined MSC’s innate and responsive fitness upon Atrazine’s exposure and can serve as an analytical model to test xenobiotics of potential environmental concerns on human MSCs. The present data derived from human MSCs further appends the knowledge about Atrazine exposure on health outcomes associated with immune homeostasis, regeneration and angiogenesis.

## Materials and methods

### Isolation and culture of human bone marrow MSCs

MSCs were derived from discarded and de-identified bone marrow filters at the end of bone marrow harvest from normal healthy donors (Age range 18-45) following IRB protocol#2016-0298 at University of Wisconsin Madison. Mononuclear cells (MNCs) were isolated from the bone marrow through a Ficoll density centrifugation gradient. Isolated MNCs were plated onto tissue culture plates containing 1x α-Minimum Essential Medium (Corning, NY, USA) with human platelet lysate (Millcreek Life Sciences, MN, USA) and penicillin/streptomycin/amphotericin B (Hyclone, UT, USA). Non-adherent suspension cells were aspirated and adherent cells washed with phosphate-buffered saline. Cells were maintained by replacing fresh media every 48-72 hours. After the observation of colonies, cells were harvested and reseeded with the density of 3000-5000 cells per centimeter square. Cells were passaged consistently with the maximum confluence of 70% to 80% and cryopreserved until needed for the experiments. MSC identity was confirmed based on cell marker expression as defined in earlier studies (CD45-CD105+CD44+CD90+CD73+) (32, 52). 48-72 hours prior to the experiment, cryopreserved MSCs are thawed and culture rescued. Experiments were performed with the culture media 1x α-Minimum Essential Medium containing fetal bovine serum and penicillin/streptomycin/amphotericin B.

### MSC exposure to Atrazine

Atrazine (TCI America, OR, USA) was prepared with a two-step process. In the first step, Atrazine was dissolved in a high concentration (100mg/ml) in Dimethyl Sulfoxide (DMSO). Subsequently, DMSO containing Atrazine was further diluted in the 1x α-Minimum Essential Medium to obtain the concentration of 1000 µg/mL and were sonicated for 30-45 minutes to increase the solubility. After sonication, Atrazine stocks were stored in -20C until needed for experiments. A similar procedure was done for DMSO without Atrazine, which served as a vehicle control. Atrazine or DMSO were thawed as needed and used with the appropriate dilutions as indicated in the experiments. MSCs seeded onto 96 well plates were exposed with the appropriate concentrations of Atrazine or vehicle. After the incubation period, cells were harvested and supernatants collected for individual assays. For the experiments with IFNγ stimulations, MSCs were exposed to appropriate concentrations of Atrazine. After the exposure period, cells were harvested, counted and reseeded into 96 well plate with the normalized cell density. Subsequently, cells were stimulated with and without human recombinant IFNγ (Gibco Thermofisher, MA, USA) for 48 hours, and the supernatants were stored, and cells were subjected to flow cytometry.

### Metabolic assay

MSCs exposed to Atrazine or vehicle were incubated with 3-(4,5-dimethylthiazol-2-yl)-2,5-diphenyltetrazolium bromide (MTT) (TCI America, OR, USA) (0.5mg/ml final concentration) for five hours. Supernatants were removed and formazan crystals were dissolved with organic solvent. Optical densities were measured at 560 nm, and background at 670nm was subtracted.

### Flow cytometry

Atrazine or vehicle exposed MSCs were harvested and stained with BD Viaprobe (BD Biosciences, USA) and acquired in BD FACS Aria II flow cytometer to quantitate percentage forward scatter high and side scatter high populations. +/-IFNγ stimulated MSC populations were stained with antibodies HLA-ABC APC (Clone W6/32) (Biolegend, USA), HLA-DR PE (Clone G46-6) (BD Biosciences, USA) along with BD Viaprobe and acquired in BD FACS Aria II flow cytometer. Mean fluorescent intensity and histogram analysis for the marker expression was performed with Flow Jo software. +/- Atrazine exposed MSCs were subjected to BD Phosflow analysis. Cells were stimulated with appropriate concentrations of IFNγ for 15 minutes and were fixed with BD Cytofix buffer (BD Biosciences, USA) for 10 minutes. After fixation, cells were permeabilized with BD Phosflow Perm III Buffer (BD Biosciences, USA) for 30 minutes and then were subjected to staining with Alexa Fluor 647 Anti-Stat1 (pY701) antibody (BD Biosciences, USA). Stained cells were acquired in BD FACS Aria II flow cytometer and the results were analyzed in Flow Jo software. For intracellular IL-8 detection, Atrazine or vehicle exposed MSCs were incubated overnight with Golgiplug (BD Biosciences, USA). Intracellular flow cytometry staining was performed with BD Cytofix and Cytoperm procedure with IL-8 APC antibody (Clone E8N1) (BD Biosciences, USA) and acquired in BD FACS Aria II flow cytometer.

### siRNA transfection on human MSCs

MSCs were seeded at a density of 10,000 cells per well in 96-well plates. Next day, cells were transfected with either non-targeting control siRNA or IL-8 SMART Pool siRNA (Horizon Discovery, CO, USA) with 1x α-Minimum Essential Medium containing 10 mM HEPES and Dharmafect transfection reagent 1. Transfection procedure was followed as defined in earlier studies and according to the manufacturer instructions (32) (Horizon Discovery, CO, USA). After five hours of transfection, cells were replaced with fresh 1x α-Minimum Essential Medium containing fetal bovine serum and penicillin/streptomycin/amphotericin B. After overnight resting, appropriate concentrations of Atrazine or vehicle was added and the cells were incubated for seven days. After seven days, supernatants were collected and stored. Cells were subjected to metabolic assays.

### Secretome assays

Supernatants derived from appropriate experimental conditions that were stored at -80°C were thawed and centrifuged at 500 x *g* for 5 minutes to remove debris. Subsequently they were subjected to magnetic bead-based multiplex according to the manufacturer instructions. Cytokine 30-Plex Human Panel (Thermofisher, USA) includes G-CSF(Granulocyte-Colony Stimulating Factor), GM-CSF (granulocyte-macrophage colony-stimulating factor), IFN-α, IFN-γ, IL-1β, IL-1RA, IL-2, IL-2R, IL-4, IL-5, IL-6, IL-7, IL-8, IL-10, IL-12 (p40/p70), IL-13, IL-15, IL-17, TNF-α, Eotaxin, IP-10, MCP-1 (Monocyte chemoattractant protein-1), MIG (Monokine induced by gamma), MIP-1α (Macrophage inflammatory protein-1 alpha), MIP-1β (Macrophage Inflammatory Protein-1 beta), RANTES, EGF (Epidermal Growth Factor), FGF-basic (Fibroblast growth factor 2), HGF (Hepatocyte Growth Factor), VEGF (Vascular Endothelial Growth Factor). Angiogenesis 16-plex panel includes Angiopoietin-1, BMP-9, CD31 (PECAM-1), EGF, EMMPRIN, Follistatin, HB-EGF, HGF, IL-8 (CXCL8), Leptin, LYVE-1, PDGF-BB, Syndecan, TIE-2, VEGF-A, VEGF-D. Results were analyzed using Luminex xMAP software to obtain the concentrations as picogramsper milliliter.

### Statistics

Data were analyzed with the GraphPad Prism 9.0 software. Linear regression analysis was performed to obtain correlation coefficient (r) and P values. Negative Logarithmic P (NLP) values were generated from the p values for conditions with wide variations. Paired t test was performed to compare the conditions from the same donor MSCs. Area Under Curve (AUC) values were obtained from each curve of the experimental conditions and were subjected to paired t test analysis. Statistical significance was confirmed with a P-value of <0.05.

## Results

### Atrazine attenuates MSC metabolic activity depending on dose and duration of exposure

**Figure 1:**
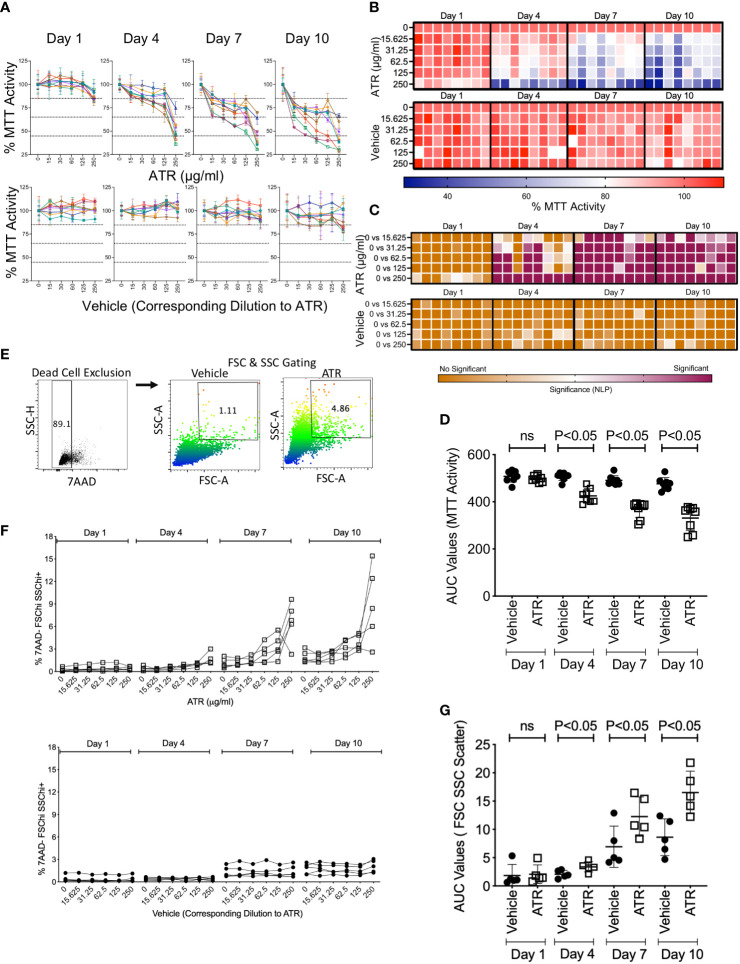
Effect of dose and duration of Atrazine exposure on the metabolic activity and scatter profile of human MSCs. Human MSCs derived from eight independent donors were seeded in 96 well plate and treated with the indicated concentrations of Atrazine (μg/ml) or respective dilutions of the vehicle DMSO. MTT assay was performed longitudinally on the indicated days, and optical density was measured. Optical density values of individual donors at each time point without Atrazine/vehicle were normalized as 100 percent and values from other concentrations were calculated as percentage MTT activity. Dose dependent effect of Atrazine or vehicle on % MTT activity is shown longitudinally at the indicated time points using **(A)** spaghetti plots and **(B)** heat map. In the heat map, each cell denotes the individual MSC donor. Each spaghetti plot curve with unique color denotes distinct MSC donor. Means and standard deviations are derived from the triplicate wells of each donor at each concentration of Atrazine or Vehicle. **(C)** Statistical significance (Negative Logarithmic of P Values (NLP)) of the percentage MTT activity is shown as a heat map for the comparison of indicated conditions of Atrazine or vehicle for all the donors. Two-Way ANOVA multiple comparison test was performed to obtain the NLP values. **(D)** Area Under Curve (AUC) values were obtained from each curve with Atrazine or vehicle at the indicated time points and were plotted with mean and standard deviation. Atrazine or vehicle exposed MSCs from five independent donors were harvested at the indicated time points and were stained with 7AAD dye to exclude dead cells. Flow cytometry was performed to determine forward scatter and side scatter profiles. **(E)** Representative flow cytometry plot and gating strategy are shown. 7AAD- (live cells) were first gated, and subsequently Forward Scatter hi and Side Scatter Hi populations were gated to identify the percentage of 7AAD- FSC^Hi^SSC^Hi^+ populations. **(F)** Dose dependent effect of Atrazine or vehicle on % 7AAD-FSC^hi^SSC^hi^+ population is shown longitudinally at the indicated time points. **(G)** AUC values of % 7AAD-FSC^hi^SSC^hi^+ were obtained from each curve with Atrazine or vehicle at the indicated time points, and were plotted with mean and standard deviation.

We have investigated dose dependent and longitudinal effect of Atrazine on human MSCs derived from the bone marrow of healthy human individuals (n=8 donors). MSC identity was confirmed based on appropriate marker expression as defined in our earlier studies (CD45−CD105+ CD44+ CD90+ CD73+) (32). MSCs derived from the passages between 2 and 5 were used on the experiments. We evaluated metabolic fitness of MSCs in the presence of various concentrations of Atrazine or vehicle (DMSO with respective dilutions) on days 1, 4, 7 and 10 utilizing MTT assay. In this assay, MSCs’ fitness in reducing the tetrazolium dye MTT to its insoluble formazan is evaluated, which is dependent on cellular energetics and NAD(P)H-dependent oxidoreductase enzymes and thus represents its metabolic activity (32). Our results demonstrated that Atrazine dose dependently inhibits the metabolic activity of MSCs derived from all the donors (**Figures 1A, B**). In addition, further statistical comparative analysis has identified that lower concentrations of Atrazine (15.625 and 31.25 μg/ml) do not reduce the metabolic activity of MSCs during a short-term (Day 1 and 4) exposure (**Figure 1C**). However, longer exposure (Day 7 and 10) with the same concentrations (15.625 and 31.25 μg/ml) have significant impact (**Figure 1C**). Comparative Area under Curve (AUC) analysis between vehicle control dilutions and Atrazine identified that vehicle controls have no effect (**Figure 1D**). Altogether this analysis has identified that Atrazine’s effect on MSCs is dependent on dose and duration of the exposure. We also tested the effect of *in vitro* culture passage (early vs late passage) on MSC’s metabolic sensitivity to Atrazine. Our results demonstrate that there is no significant difference between early and late passage MSC’s sensitivity to Atrazine (**Figure S1**).

### Atrazine exposure increases size and granularity of human MSCs

We investigated phenotypical changes induced by Atrazine on MSCs. MSCs derived from 5 independent donors were exposed with various concentrations of Atrazine or vehicle. Cells were harvested longitudinally at regular intervals and stained for viability dye 7AAD. Subsequently forward scatter and side scatter profiles were analyzed under flow cytometry which indicate their size and granularity. To exclude dead/apoptotic cells, we specifically gated on 7AAD negative populations which includes only live cells in the analysis (**Figure 1E**). Thus, this assay system evaluates the effect of Atrazine or vehicle exposure in modulating the size and granularity of live but not dead/apoptotic cells. Our results indicate that Atrazine dose dependently induces high scatter profile (Size and Granularity) on live MSCs (**Figures 1F, G**). We also identified that induction of high scatter profile on MSCs is also dependent on duration of the exposure (**Figures 1F, G**). These effects are absent in vehicle exposed conditions (**Figures 1F, G**). These results identify Atrazine induced non-toxic cellular phenotypic alterations of MSCs. In addition, these results further demonstrate that Atrazine induced high scatter properties on MSCs are dependent on dose and duration of the exposure.

### Atrazine differentially modulates the innate secretome of human MSCs

**Figure 2:**
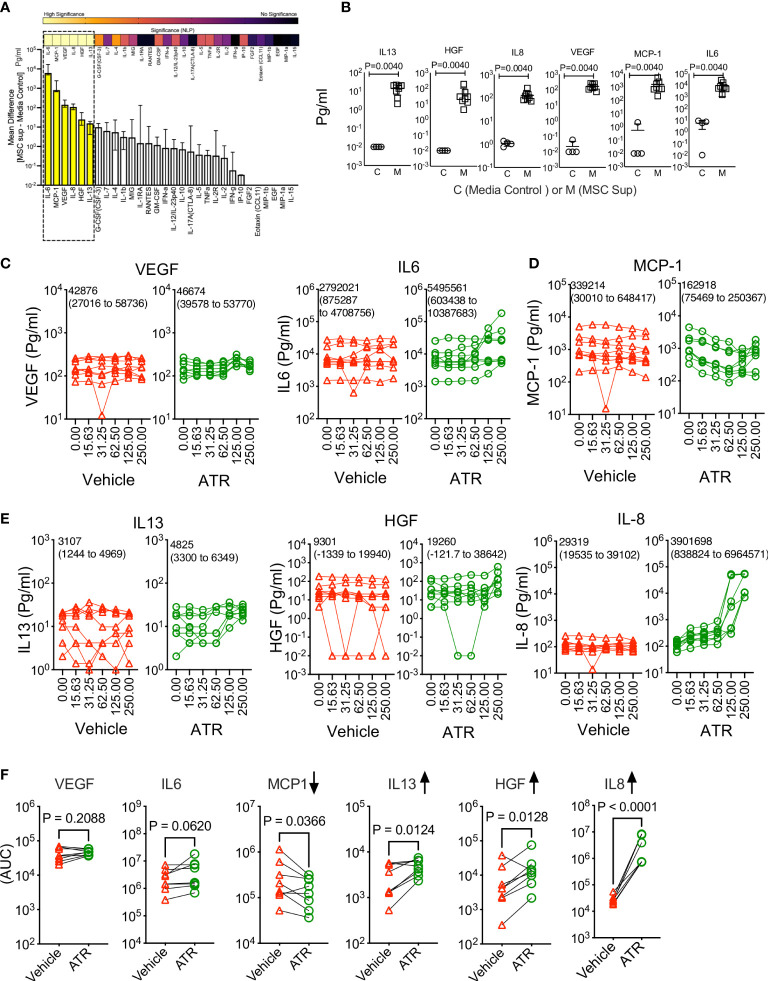
Atrazine exposure on the innate secretory fitness of human MSCs. Human MSCs derived from eight independent donors were seeded in a 96 well plate and treated with the indicated concentrations of Atrazine (μg/ml) or respective dilutions of the vehicle DMSO. After seven days of culture, supernatants were collected and analyzed for 30-plex secretome using Luminex™ xMAP (multi-analyte profiling) technology. **(A)** MSCs’ innate secretome in the absence of Atrazine or vehicle is plotted with 30plex analysis. Secretory levels of each analyte from MSC cultures (MSC Supernatant) are subtracted from control media (without MSCs) and are plotted hierarchically based on their secretion levels. Statistical significance (Negative Logarithms of P value) of these secretions is also shown as a heat map. **(B)** Statistically significant secretory molecules of MSCs, derived from eight independent donors, are individually plotted. MSC Sup= MSC supernatant and Control=Control media without MSCs. **(C–E)** Dose dependent effect of Atrazine or vehicle on the statistically significant secretory molecules **(B)** are plotted. Mean AUC value derived from eight independent donors and the respective 95% confidence interval are shown within each plot. Secretory molecules are categorized as **(C)** unmodulated, **(D)** downregulated and **(E)** upregulated based on their response to Atrazine. **(F)** AUC values of individual secretory molecules, derived from the dose dependent curves of atrazine and vehicle, are plotted. Each pair of AUC values (Atrazine and vehicle) is derived from the individual donor MSCs. Paired t test analysis was performed to identify p values. Upregulated and downregulated secretory molecules are marked with appropriate arrow symbols.

We investigated the effect of Atrazine in modulating MSC’s innate fitness by analyzing their secretome panel consisting of immunomodulatory and regenerative secretory factors (**Figures 2A, B**). Supernatants collected from MSC cultures (derived from 8 independent donors) were analyzed for comprehensive 30-plex human analyte panel which includes cytokines, chemokines and growth factors. Our results demonstrate that MSCs innately secrete at least six soluble analytes (IL-6, MCP-1, VEGF, IL-8, HGF, IL-13) that are significantly detected in MSC culture supernatants over the control media (**Figures 2A, B**). Next, we investigated the effect of Atrazine or vehicle (7 days exposure) in modulating these six innate secretory molecules of MSCs. To determine Atrazine’s dose dependent effect, AUC values of individual secretory molecules were determined with Atrazine and vehicle conditions for each MSC donor. Three patterns of innate secretome are identified. (1) AUC values of VEGF and IL-6 are not significantly different between Atrazine and vehicle exposure which indicates that both of these secretory molecules are not affected by Atrazine’s exposure (**Figures 2C, F**). (2) Atrazine significantly (P<0.05) inhibits MCP-1 secretion by MSCs as identified by their reduction in AUC values compared to the vehicle (**Figures 2D, F**). (3) Secretion of IL-13, HGF and IL-8 by MSCs are significantly (P<0.05) upregulated by Atrazine as identified with their increased AUC values compared to the vehicle control (**Figures 2E, F**). Specifically, upregulation of IL-8 is robust and substantial with high statistical significance (P<0.0001) (**Figures 2E, F**). The other secretory molecules (24 molecules) that are not innately secreted by MSCs are not modulated by Atrazine exposure (**Figure S2**). These results have demonstrated that Atrazine exposure not only downregulate but also upregulate and unmodulate the secretome of MSCs.

### Atrazine-induced IL-8, metabolic activity, and secretory molecules of MSCs

**Figure 3:**
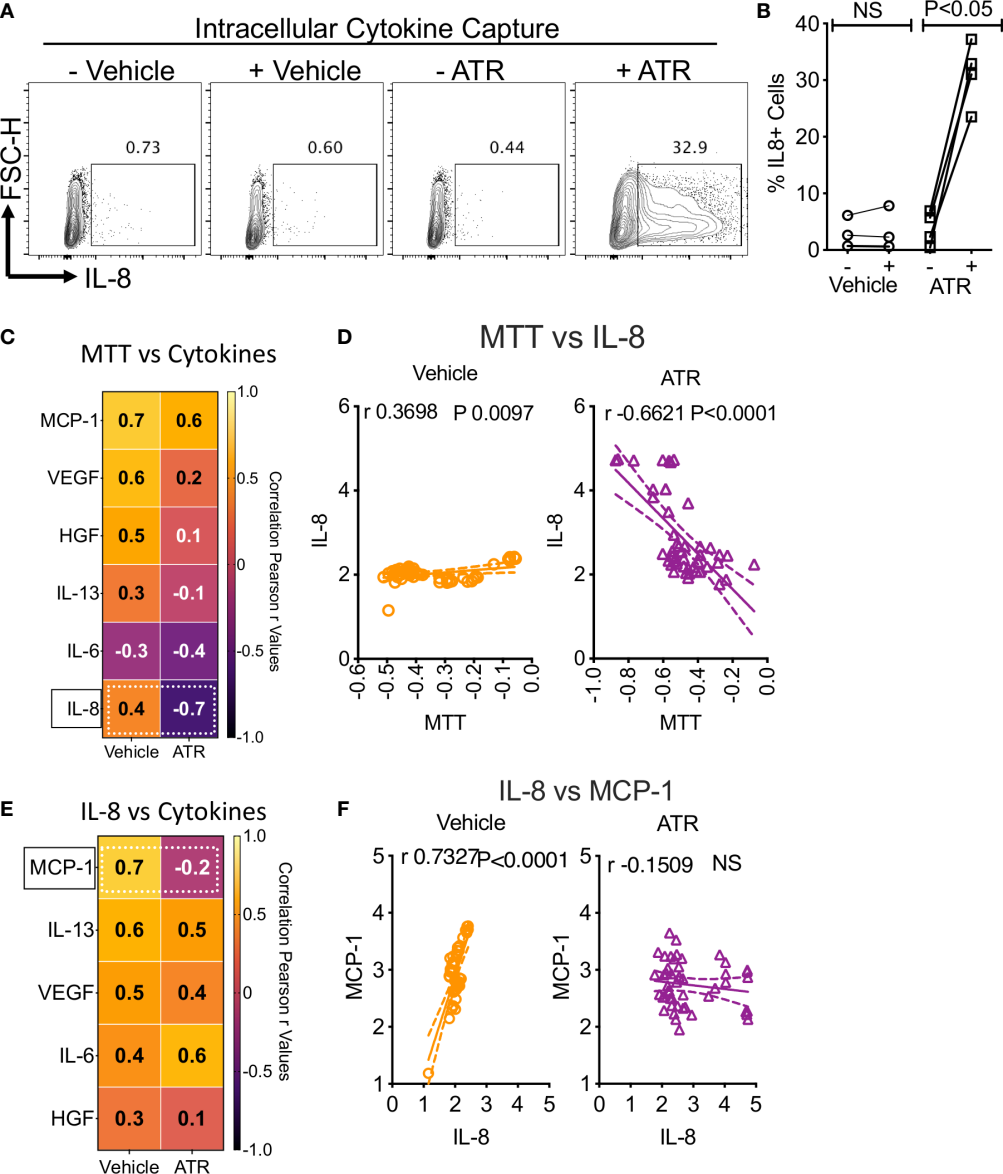
Dynamics of Atrazine induced IL-8 on MSCs. Atrazine or vehicle exposed MSCs were treated with Golgi transport inhibitor to capture intracellular accumulation of IL-8. Intracellular cytokine staining was performed to identify IL-8 expression through flow cytometry. **(A)** Representative flow cytometry plot and gating are shown to identify IL-8 expression in Atrazine and vehicle treated cells. **(B)** Cumulative IL-8 expression with MSCs from four independent donors is shown with statistical significance. **(C)** Optical density values of MTT metabolic assays, derived from Atrazine and vehicle exposure conditions, were subjected to linear regression analysis with the levels of corresponding individual secretory molecules. Correlation coefficient (r) values are plotted as a heat map. The major shift from direct to inverse correlation of IL-8 and MTT metabolic activity in the conditions of vehicle and Atrazine is shown with the dotted line box. **(D)** Linear regression plots and statistical significance show the correlation between IL-8 secretion and MTT metabolic activity in vehicle and Atrazine exposure conditions. MTT assay values (optical density) and IL-8 concentrations (pg/ml) were transformed to logarithmic data to fit the regression line. **(E)** Quantitative levels of IL-8 were subjected to linear regression analysis with other secretory molecules derived from the same culture conditions with Atrazine or vehicle exposure. Correlation coefficient (r) values are plotted as a heat map. The major shift from direct to inverse correlation of IL-8 and MCP-1 in the conditions of vehicle and Atrazine is shown with the dotted line box. **(F)** Linear regression plots and statistical significance show the correlation between IL-8 and MCP-1 secretion in vehicle and atrazine exposure conditions. IL-8 and MCP-1 concentrations (pg/ml) were transformed to logarithmic data to fit the regression line. r values of 1 and -1 indicate the best direct and inverse correlations respectively, while 0 indicates no correlation.

To further confirm Atrazine-induced IL-8 secretion in human MSCs, we performed intracellular cytokine capturing analysis using flow cytometry (n=4 independent donors, Atrazine (250μg/ml) exposure for 7 days). Our results demonstrate that Atrazine but not vehicle exposure induces IL-8 expression in human MSCs as identified by their intracellular accumulation captured with Golgi transport blockage (**Figures 3A, B**). Next to identify the relationship between Atrazine induced IL-8 secretion and MSC’s metabolic activity, we performed a linear regression analysis between MSC’s MTT activity and their six innate secretory molecules (**Figure 3C**). These analyses identified the strongest inverse correlation (r value closer to -1.0) between MTT activity and IL-8 secretion with Atrazine but not vehicle exposure (**Figures 3C, D**). Next, we investigated the relationship between Atrazine induced IL-8 secretion and other innate secretory molecules (**Figure 3E**). Linear regression analysis between the secretion levels of IL-8 and other secretory molecules (IL-6, IL-13, HGF, VEGF, MCP-1) identified that IL-8 exhibits the strongest direct correlation (r value closer to 1.0) with MCP-1 which is abolished upon Atrazine exposure (**Figures 3E, F**). These results suggest that Atrazine induced IL-8 display a unique relation with MSC’s metabolic activity and MCP-1 secretion.

### Atrazine attenuates IFNγ-induced upregulation of MHC Class II but not MHC Class I and STAT-1 phosphorylation on MSCs

**Figure 4:**
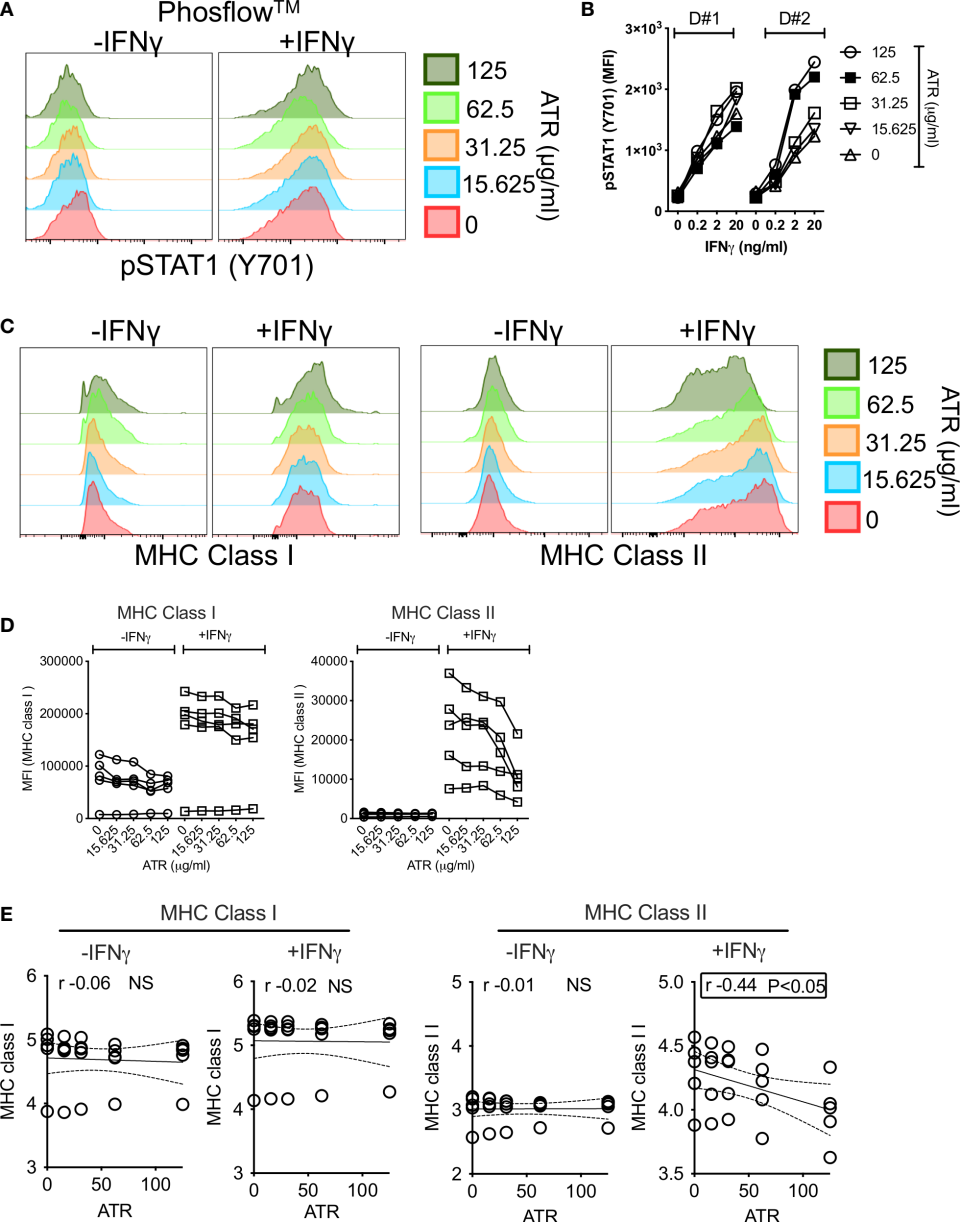
Effect of Atrazine exposure on IFNγ induced early phosphorylation and downstream surface molecules on human MSCs. MSCs exposed with various concentrators of Atrazine were stimulated with IFNγ for 15 minutes. Phosflow technology was deployed to detect STAT1 phosphorylation (Y701) using flow cytometry. **(A)** Representative histogram overlay plots are shown for IFNγ induced pSTAT-1 expression on MSCs exposed with various concentrations of Atrazine. **(B)** Cumulative Mean Fluorescent Intensity (MFI) values of pSTAT-1 from two independent donor MSCs exposed to varying concentrations of Atrazine are shown. Indicated concentrations of IFNγ stimulation was performed in these conditions. **(C)** MSCs exposed to varying concentrations of Atrazine were stimulated with 0 or 20ng/ml IFNγ for 48 hours, subsequently stained with the antibodies to MHC Class I and MHC Class II surface markers, and acquired in flow cytometry. Representative histogram overlay plots are shown for MHC Class I and MHC Class II expression. **(D)** +/- IFNγ induced MFI values of MHC Class I and MHC Class II from five independent donor MSCs exposed to varying concentrations of Atrazine are cumulatively shown. **(E)** MFI values of MHC Class I and MHC Class II molecules were subjected to linear regression analysis with Atrazine exposure concentrations to identify their correlations. Statistically significant inverse correlation between IFNγ induced MHC Class II and Atrazine exposure is highlighted with the box.

MSCs’ responsive fitness to exogenous stimulation, such as IFNγ, is considered as the surrogate measure of their immune functionality (53, 54). Hence, we aimed to investigate the effect of Atrazine exposure on IFNγ induced upstream signaling activation and downstream key immune effector molecules on MSCs. IFNγ induces JAK-STAT signaling pathway on MSCs through the phosphorylation of STAT1 (pSTAT1 at Tyr701) (33). We deployed Phosflow™ technology to capture STAT1 phosphorylation on +/- Atrazine exposed MSCs. +/-Atrazine exposed MSCs (n=2 independent donors, +/- Atrazine exposure with various concentrations around 10 days) were stimulated with IFNγ for 15 minutes and STAT1 phosphorylation at Tyr701 was analyzed using Phosflow™ technology. Our results indicate that IFNγ phosphorylates STAT1 on MSCs irrespective of Atrazine exposure at similar levels (Area under curve (AUC) of pSTAT1 MFI with each concentration of atrazine from two independent donors (cumulatively) did not show a statistical significance) which suggests that Atrazine does not attenuate IFNγ induced upstream signaling on MSCs (**Figures 4A, B**). Next, we investigated the effect of IFNγ on downstream key immune effector molecules, MHC class I and MHC class II. MSC (n=5 independent donors) populations were exposed with various concentrations of Atrazine around 10 days and subsequently they were activated without and with IFNγ (20ng/ml) for 48 hours. Consistent with previous publications, our results also demonstrate that IFNγ upregulates both MHC Class I and MHC class II molecules on MSCs (55). Atrazine exposure does not have any effect on IFNγ dependent upregulation of MHC class I molecules on MSCs (**Figures 4C–E**). However, Atrazine attenuates IFNγ induced MHC class II molecule upregulation on MSCs (**Figures 4C–E**). Altogether these results suggest that Atrazine blocks IFNγ induced upregulation of MHC Class II but not MHC Class I and STAT1 phosphorylation on MSCs.

### Atrazine attenuates IFNγ-mediated immunomodulatory secretome responses of MSCs

**Figure 5:**
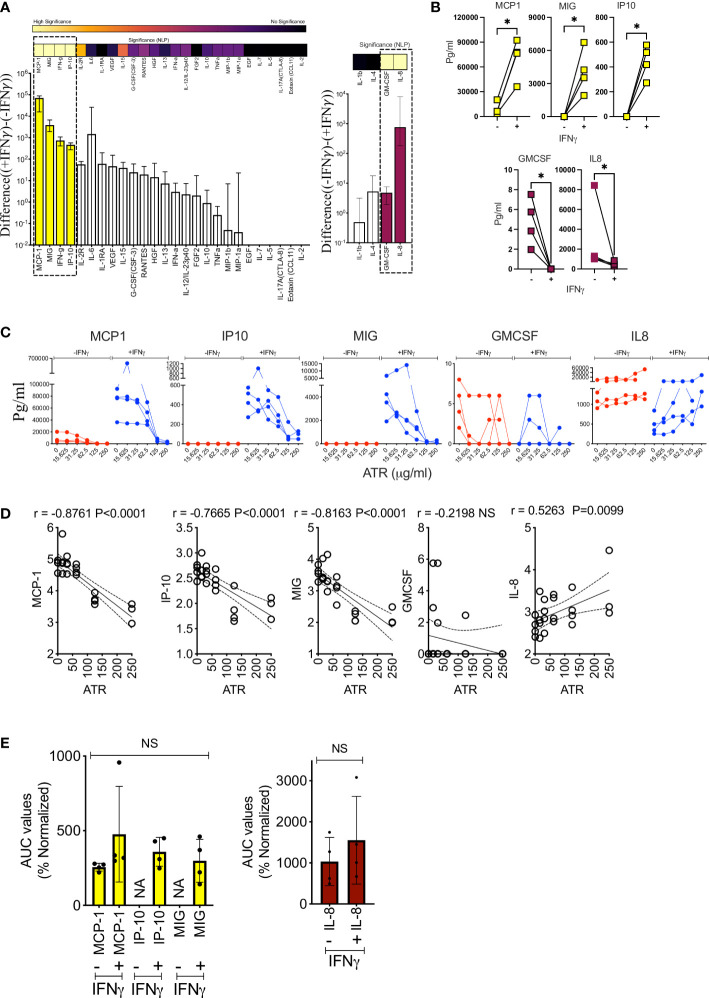
Atrazine attenuates of IFNγ responsive secretome of human MSCs. MSCs derived from four independent donors were exposed to varying concentrations of Atrazine for ten days. After harvesting, cells were seeded with normalized density and subsequently stimulated with 0 or 20ng/ml IFNγ for 48 hours. Supernatants were analyzed for 30-plex secretome using Luminex™ xMAP (multi-analyte profiling) technology. **(A)** IFNγ responsive MSCs’ secretome in the absence of Atrazine or vehicle is plotted. Difference in the secretory levels of each analyte between + and - IFNγ stimulation is hierarchically plotted. Statistical significance (Negative Logarithms of P value) of difference in secretions also shown as a heat map. **(B)** Statistically significant up or downregulated secretory molecules upon IFNγ stimulation of MSCs, derived from four independent donors, are individually plotted. **(C)** Dose dependent effect of Atrazine exposure on statistically significant IFNγ responsive secretory molecules **(B)** are plotted. **(D)** IFNγ modulated secretion levels of MCP-1, IP10, MIG, GMCSF and IL-8 were subjected to linear regression analysis with Atrazine exposure concentrations to identify their correlations. Inverse and direct correlations between IFNγ induced molecules and Atrazine exposure are shown with correlation coefficient (r) values and statistically significance. **(E)** AUC values of MCP-1, IP10, MIG, GMCSF and IL-8 derived from Atrazine dose dependent curves were normalized. Percentage normalization was calculated based on the levels of these analytes without Atrazine. Upregulated analytes MCP-1, IP10, MIG and down regulated analytes IL-8 and GMCSF are separately plotted. Ordinary One-Way ANOVA multiple comparisons are performed to determine statistical significance. NS, No significance; NA, Not Applicable. *, P<0.05.

We have investigated the effect of IFNγ induced immunomodulatory secretome on Atrazine exposed MSCs. MSC (n=4 independent donors) populations were exposed with various concentrations of Atrazine around 10 days and subsequently they were activated without and with IFNγ (20ng/ml) for 48 hours. 30-plex secretome analysis of +/- IFNγ activated MSCs (n=4 independent donors) has identified three patterns of secretome which includes IFNγ induced upregulation, downregulation and unmodulation (**Figure 5A**). IFNγ upregulates MCP-1, MIG and IP10 while GMCSF and IL-8 are downregulated (**Figure 5B**). Of these MCP-1 and IL-8 are part of MSCs’ innate secretome whereas IP10 and MIG are absent in resting MSCs which are upregulated *de novo* upon IFNγ stimulation. This 30-plex analysis also identified IFNγ in the supernatants of IFNγ stimulated MSCs which is the inoculum used for the stimulation, and hence, we did not further include in the analysis (**Figure 5A**). The rest of the other secretory molecules are neither upregulated nor down regulated by IFNγ (**Figure 5A**). Our results also show that Atrazine exposed MSCs display attenuated upregulation of MCP-1, IP-10 and MIG upon IFNγ stimulation (**Figure 5C**). Conversely, IFNγ mediated downregulation of IL-8 secretion is dose dependently relieved by Atrazine exposure (**Figure 5C**). We also observed that IFNγ mediated downregulation of GMCSF is not significantly relieved upon Atrazine exposure. Linear regression analysis between Atrazine and IFNγ responsive secretome of MSCs has also identified that Atrazine exposure predominantly correlates with secretory molecules in an inverse manner (MCP-1 (r=-0.9 P<0.0001) exhibits the top inverse correlation) except for IL-8 (**Figure 5D**). These results suggest that Atrazine exposure counteracts IFNγ responsive secretome of MSCs. To identify the relative degree of Atrazine’s effect on individual IFNγ responsive molecules, we have determined the percentage normalized AUC values, since the secretion levels of each molecule differ due to donor variability. These analyses identify that normalized AUC values of Atrazine downregulated MCP1(+/-IFNγ), IP-10(+IFNγ), MIG(+IFNγ) and upregulated IL-8(+/-IFNγ) are not significantly different (**Figure 5E**). These suggest that Atrazine exposure equitably affects +/- IFNγ responsive matrix secretome of MSCs with features of upregulation and downregulation.

### Atrazine exposure modulates the secretion of MSC’s angiogenic factors

**Figure 6:**
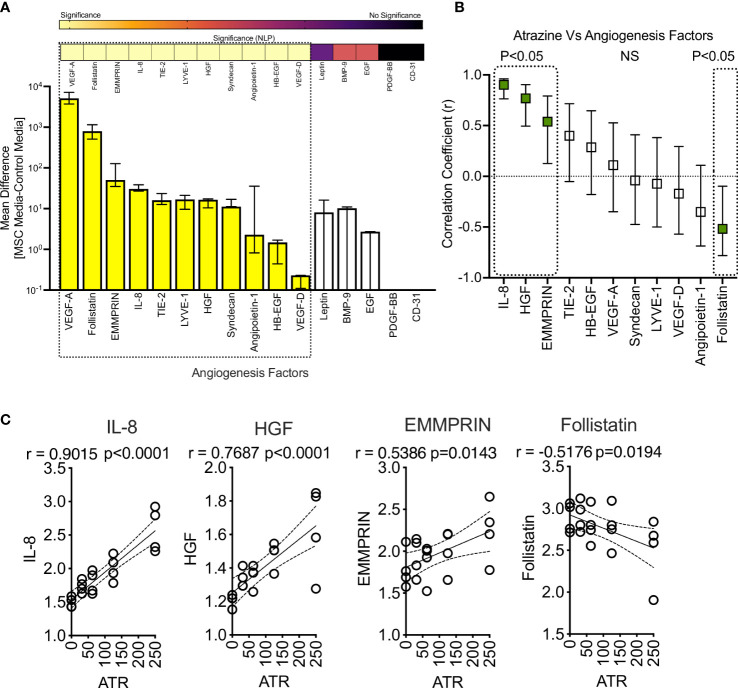
Effect of Atrazine exposure on angiogenic secretory factors of human MSCs. Human MSCs derived from four independent donors were seeded in a 96 well plate and treated with various concentrations of Atrazine. After seven days of culture, supernatants were collected and analyzed for 16-plex angiogenic secretory factors using Luminex™ xMAP (multi-analyte profiling) technology. **(A)** MSCs’ angiogenic secretory factors in the absence of Atrazine or vehicle is plotted. Secretory levels of each analyte from MSC cultures are subtracted from control media (without MSCs) and are plotted hierarchically based on their levels of secretion. Statistical significance (Negative Logarithms of P value) of these secretions also shown as a heat map. **(B)** Secretion levels of statistically significant angiogenic factors were subjected to linear regression analysis with Atrazine concentrations to identify their correlations. Hierarchical ranking of correlation coefficient (r) values with 95% confidence intervals for each angiogenic molecule is shown in the scale of -1 to + 1 which indicates the best inverse to direct correlations respectively. Statistically significant r values (P<0.05) are marked with dotted boxes. Non-Significant (NS) correlations are also shown. **(C)** Individual linear regression plots with statistical significance are shown for IL-8, HGF, EMMPRIN and Follistatin.

IL-8 is a key factor that plays a major role in the angiogenesis of blood vessels (56). MSCs have the propensity to drive angiogenesis as part of their repair and regenerative properties (57, 58). Since Atrazine upregulates IL-8 secretion on MSCs, we further investigated the effect of Atrazine on other angiogenic factors of MSCs. MSCs (derived from 4 independent donors) were cultured with various concentrations of Atrazine for 7 days. Supernatants collected from MSC cultures were analyzed for comprehensive 16-plex human analyte panel focusing on angiogenic factors using Luminex xMAP™ technology. Our results demonstrate that MSCs secrete at least 11 soluble analytes (VEGF-A, Follistatin, EMMPRIN, IL-8, TIE-2, LYVE-1, HGF, Syndecan, Angiopoietin-1, HB-EGF, VEGF-D) that are significantly detected in the supernatants over the control media though their individual secretion levels vary (**Figure 6A**). Next, we investigated the effect of Atrazine exposure on these 11 angiogenic factors. Correlation analysis of individual angiogenic factors and Atrazine concentrations have identified at least three independent relationships which includes direct, indirect and no correlations (**Figure 6B**). IL-8, HGF, and EMMPRIN exhibit significant direct correlations which indicates Atrazine upregulates their secretion (**Figures 6B, C**). In contrast, Follistatin exhibits an inverse correlation which indicates its downregulation upon Atrazine exposure (**Figures 6B, C**). At least seven angiogenic molecules do not exhibit any significant correlation with Atrazine exposure (**Figure 6B**). Altogether, these results signify the dichotomy of Atrazine’s effect in modulating the angiogenic factors of MSCs.

### MSC sensitivity to Atrazine mediated inhibition of metabolic activity is not dependent on IL-8

**Figure 7:**
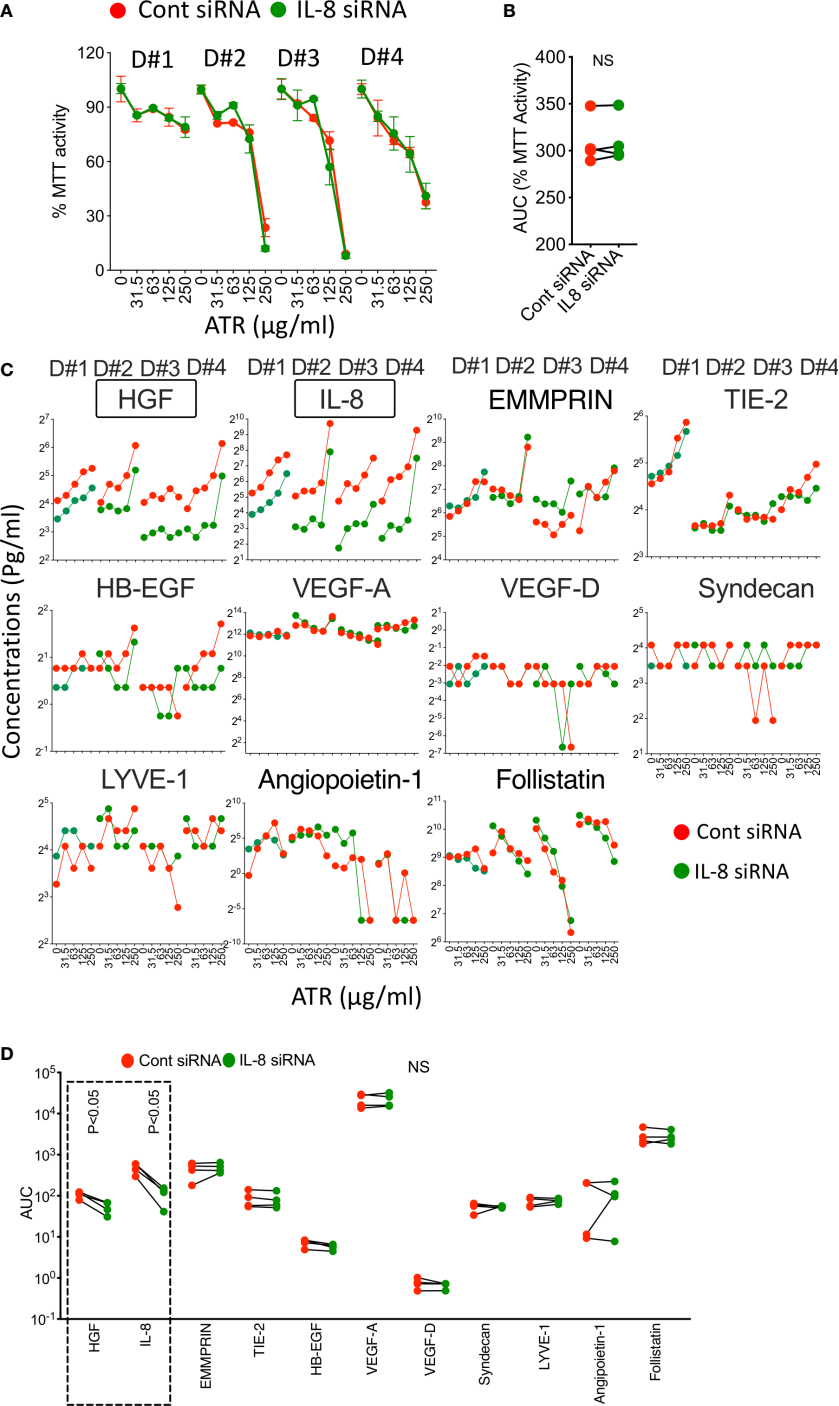
Role of Atrazine induced IL-8 on MSC’s metabolic activity and secretion of angiogenic factors. Control or IL-8 siRNA silenced human MSCs derived from four independent donors were subjected to Atrazine exposure for seven days. Supernatants were stored, and MTT assays were performed with the cells. **(A)** Dose dependent effect of Atrazine on % MTT activity is shown for each MSC donor (D#1, D#2, D#3, D#4). **(B)** AUC values of MTT activity derived from the curves of control and IL-8 silenced MSCs are shown. Paired t test was performed to identify statistical significance. **(C)** Dose dependent effect of Atrazine on the secretion of angiogenic factors from control or IL-8 silenced MSCs of four independent donors (D#1, D#2, D#3, D#4) is shown. **(D)** AUC values of angiogenic factors derived from the curves of control and IL-8 silenced MSCs are shown. Paired t test was performed to identify statistical significance. Statistically significant (P<0.05) changes (IL-8, HGF) between control and IL-8 silenced conditions are marked with dotted box. NS, Non-significant.

Since IL-8 secretion is substantially upregulated on MSCs upon Atrazine, we aimed to identify if Atrazine induced IL-8 secretion also plays any role on MSC’s metabolic activity and sensitivity to Atrazine as a loop mechanism. We performed siRNA knockdown of IL-8. Control or IL-8 silenced MSCs (4 independent donors) were exposed with differential concentrations of Atrazine, and MTT activity was measured eight days post exposure. Our results demonstrate that both IL-8 and control silenced MSCs are equally susceptible to Atrazine mediated dose dependent inhibition of metabolic activity. Further analysis has identified that both IL-8 and control silenced MSCs do not significantly differ in their AUC values (**Figures 7A, B**). These results suggest that Atrazine induced IL-8 has no effect on MSC’s metabolic sensitivity to Atrazine. Previous studies have shown that human MSCs sense xenobiotics such as 2,3,7,8-Tetrachlorodibenzo-p-dioxin (TCDD) sense through Aryl Hydrocarbon Receptor (AHR) (59). However, our results show that knockdown of AHR on MSCs do not modulate MSC’s sensitivity to Atrazine (**Figure S3**) which suggests that AHR does not play a role in the MSC and Atrazine axis.

### Knockdown of Atrazine induced IL-8 secretion on MSCs reduces certain angiogenic factors

Next, we aimed to identify if Atrazine induced IL-8 also modulates other angiogenic factors. We evaluated the supernatants of control and IL-8 siRNA silenced MSCs that were exposed (7 Days) with various concentrations of Atrazine for 11 angiogenic factors using Luminex xMAP technology (**Figure 7C**). Our results demonstrate that IL-8 silenced MSCs secrete lower levels of IL-8 (as identified with statistically significant lower AUC values) compared to control silenced MSCs (**Figures 7C, D**). In addition, IL-8 silenced MSCs also reduce HGF secretion which suggests that Atrazine induced IL-8 regulates HGF secretion on MSCs (**Figures 7C, D**). No significant modulation is observed in other angiogenic factors when comparing IL-8 and control silenced MSCs (**Figures 7C, D**). Altogether these results suggest that Atrazine induced IL-8 enhances specific angiogenic factors such as HGF but not others (**Figures 7C, D**).

## Discussion

Identifying the outcome measures of MSCs upon exposure with xenobiotics has been a challenge due to the complexity of MSCs’ mechanism of action and their wide array of properties. Here, we have deployed an assay matrix technology that captures more than a single functionality of MSCs which cumulatively identified dysfunctional and functional effector pathways upon Atrazine exposure.

Environmental significance and health effects of Atrazine vary depending on the dose and duration of their exposure. Wind transportation and run off waters are the principal modes of Atrazine exposure from agricultural and residential garden usage to human population (35–37). Thus, the degree of exposure and environmental availability varies from time to time depending on the usage of Atrazine. Generally, pesticides are categorized based on their threshold exposure concentrations as poison (I), moderately toxic (II), slightly toxic (III) and relatively nontoxic (IV). These threshold concentration values also vary depending on the route of exposure as oral, dermal and inhalation (60). Chronic exposure is further complex since the exposure can occur with sporadical repetition or continuously throughout one’s life time. Atrazine is considered as a category III herbicide/pesticide, and hence its dose and duration of exposure can make an impact (34). In an attempt to address this, we exposed MSCs from different donors to a broad range of Atrazine concentrations with a differential incubation period. Our results have shown that lower concentrations and shorter duration of Atrazine exposure do not have any impact on MSCs, although, this changed over a prolonged exposure. Our results also show that Atrazine not only modulates the metabolic activity of MSCs in a dose and time dependent manner, but also their size and granularity (cellular hypertrophy). Specifically, we have excluded dead cells in the size and granularity analysis which suggests that non-toxic concentrations of Atrazine cause hypertrophy of MSCs which is a phenomenon often seen with cellular abnormality. Altogether, acute or chronic exposure of Atrazine which is associated with variations in environmental conditions, such as wide range of concentrations and duration could potentially impact human health.

World Health Organization sets the limit for atrazine in drinking water as 100 parts per billion (100μg/L). However, it also needs to be emphasized that in the rural communities, closer to agricultural lands with atrazine application, the drinking water from private wells are not monitored for chemical contaminants. Stradtman et al. discussed the challenges and complications in translating precise human exposure of Atrazine to laboratory studies due to limitations in accurate drinking water monitoring data and biomonitoring assessments (61). Previous *in vitro* exposure studies have used a wide range of Atrazine concentrations in different cell lines. Atrazine exposure was tested in Pheochromocytoma (PC12) cells *in vitro* with a concentration ranging from 0-200μM (0 to 43μg/ml) (62). Dose dependent effect of Atrazine (0 to 100μg/ml) is shown in RAW264.7 cells (63). Neurodevelopment toxicity of atrazine is shown in an *in vitro* human embryonic stem cells based neural differentiation model with a dose dependent concentration from 0 to 500μM (0 to 108 μg/ml) (64). Atrazine mediated induction of apoptosis in SH-SY5Y human neuroblastoma cells were demonstrated with a concentration ranging from 0 to 50ug/ml (65). Toxic effects of atrazine are demonstrated in Chinese Hamster Ovary (CHO-K1) cell line with a concentration ranging from 10–160 μg/mL (66). In the present study, we have demonstrated the dose dependent effect of atrazine on human MSCs with a wide concentration ranging from 0–250 μg/mL with differential exposure duration. Thus, the present results add to the existing data on the potential health effects of atrazine with a relation to regenerative human MSCs.

Our assay matrix technology incorporates two strategies to define the effect of Atrazine on human MSCs. In the first strategy, MSC’s fitness in secreting more than a single secretory molecule is concomitantly investigated as a measure of its innate functionality. We identified that only a few, but not all, secretory molecules are dose dependently modulated (either upregulated or down regulated) upon Atrazine exposure. MCP-1 derived from MSCs is shown to play a significant role in immune modulation and tissue regeneration (67–69). Considering the attenuation of MCP-1 secretion, MSCs are dysfunctional in executing this effector pathway. Conversely, we also identified that the secretion of IL-13, HGF and IL-8 are further upregulated upon Atrazine exposure which indicates the induction of MSC’s innate activity through these effector pathways. These shifts in MSC’s innate secretome provide insights that Atrazine exposure dose dependently cause homeostatic and physiological imbalance of MSCs from their innate and quiescent state.

In the second strategy, we identified MSC’s responsive fitness to exogenous cues. Here effector molecules of MSCs, which are evoked by exogenous cue, are enumerated and quantified as a measure of MSC’s responsive fitness. We deployed three independent analytical systems to capture the effect of Atrazine exposure on MSC’s responsive fitness. In the first system, MSC’s immediate response to exogenous cue, IFNγ, was analyzed using Phosflow™ technology. Previous study has demonstrated that quantitation of STAT1 phosphorylation on MSCs immediately upon exogenous stimulation can serve as a measure of MSC’s functionality (33). Utilizing this strategy, our results have demonstrated that Atrazine exposure does not attenuate IFNγ induced immediate STAT-1 phosphorylation on MSCs. This suggests that Atrazine exposure does not affect the immediate responsive functionality of MSCs to exogenous stimulation. In the second analytical system, the effect of Atrazine exposure on IFNγ activated cell surface molecules, MHC class I and class II, was investigated. Although MHC class I upregulation is not modulated, we observed defect in MHC class II upregulation. This corroborates with our innate secretome fitness analysis that Atrazine exposure affects only specific secretory molecules of MSCs. Previous studies have shown that defect in MHC class II expression is associated with cellular senescence or aging of MSCs (70). Our results provide insights that Atrazine exposure cause premature aging of MSC populations. In the third analytical system, we investigated the effect of Atrazine exposure on IFNγ induced secretome of MSCs. Atrazine exposure inhibits IFNγ induced secretion of MCP-1, IP10 and MIG. Conversely, Atrazine exposure also increases IFNγ repressed secretion of IL-8. MSCs’ immune plasticity due to their differential responses to exogenous cues is a key feature for its regenerative functions (53, 71, 72). The confounding effect of Atrazine on IFNγ modulated MCP-1, IP10, MIG and IL-8 provide insights that Atrazine exposure alters the immune plasticity status of MSCs. Altogether, the tripartite analytical systems in the assay matrix have identified that Atrazine affects only a certain but not all the effector pathways associated with MSC’s responsive fitness.

Angiogenic pathway focused secretome assay has identified that Atrazine exposure upregulates at least three angiogenic factors including IL-8, HGF and EMMPRIN while Follistatin is down regulated. These differential effect of Atrazine on the angiogenic factors have further confirmed that the individual outcome responses of MSCs to Atrazine exposure are not identical. Identification of this phenomenon further attests the significance of assay matrix technology in determining the effect of xenobiotic exposure on MSCs’ functionality.

IL-8 is identified as a top upregulated secretory molecule upon Atrazine exposure which showed inverse relationship with MSC’s metabolic activity and other secretory molecules, particularly MCP-1 with highest significance. Both IL-8 and MCP-1 play significant roles in angiogenesis and chemotaxis of immune cells to the site of injury and inflammation (56, 73). In the absence of Atrazine exposure, IL-8 and MCP-1 do exhibit a strong direct correlative secretion which suggests their synergistic effects in MSC’s physiological roles. However, inversion of IL-8 and MCP-1 correlation upon atrazine exposure, suggests that Atrazine alters the equilibrium of MSC’s physiological effector molecules.

Mechanistic analysis has further demonstrated that silencing of Atrazine induced IL-8 does not modulate MSC’s viability/metabolic activity. This suggests that increased IL-8 secretion is the byproduct of MSC’s response to Atrazine, and IL-8 has no role on MSC’s metabolic sensitivity to Atrazine. In addition, these data suggest that Atrazine’s modulatory effects on IL-8 secretion and metabolic activity are separable functions. However, Atrazine exposure on IL-8 silenced MSCs not only reduces IL-8 secretion but also HGF while other angiogenic factors are not affected. Thus, although IL-8 secretion is interlinked with HGF, Atrazine exposure does modulate many other angiogenic factors. Altogether assay matrix technology confirm that the mechanism of action of Atrazine on MSCs is specific and tightly regulated without affecting all the characteristics of MSCs. The present study focuses on MSCs derived from the bone marrow of healthy individuals as a model while assay matrix technology can be adopted to test MSCs derived from other tissue sources.

Testing of xenobiotics on regenerative MSC populations has increasingly being recognized. Recent work by Behan Bush et al. demonstrated the toxicity of xenobiotics Aroclor and Non-Aroclor mixtures of Polychlorinated Biphenyl exposure on Human Adipose MSCs (74). Another recent study has demonstrated that 2,3,7,8-tetrachlorodibenzo-p-dioxin alters essential transcriptional regulators of osteogenic differentiation in bone marrow MSCs (75). Our present study further strengths this concept by molecular profiling of MSCs in their innate/native (Metabolic activity, Immunomodulatory secretome, Angiogenic secretome) and responsive functionality (Exogenous IFNγ induced secretome, cell surface molecules, phosphorylation signals) post Atrazine exposure. Future studies are warranted in defining further impact of Atrazine on other MSC functionalities including but not limited to their differentiation, hematopoietic support, tissue repair and innate and adaptive immune systems. It is also of future interest to study the effect of Atrazine on other visceral tissue derived MSCs such as adipose and umbilical cord derived MSCs. In the present study we have utilized MTT assay as a measure of MSC’s viability and metabolic activity. However additional metabolic assays with higher detection sensitivity representing the individual bio metabolic pathways of MSCs needs to be developed in assessing the impact of atrazine.

Our study showed that Atrazine may be an important environmental factor in altering the immunomodulatory potential of MSCs in some specific conditions. This provides insights on the consequences of herbicide exposure on both acute and long-term health effects. Assay matrix technology can be utilized to evaluate the exposure of environmental xenobiotics on the innate and responsive fitness of MSCs which informs the cumulative functionality of MSCs.

## Data availability statement

The original contributions presented in the study are included in the article/**Supplementary Material**. Further inquiries can be directed to the corresponding author.

## Ethics statement

MSCs were derived from discarded and de-identified bone marrow filters at the end of bone marrow harvest from normal healthy donors in accordance with IRB exemption #2016-0298 “Collection of discarded bone marrow and peripheral blood cell bag products” at the University of Wisconsin Madison. UW-IRB waived the requirement of written informed consent for participation.

## Author contributions

CU and BP performed majority of the experiments related to MSCs and Atrazine. CU drafted a portion of the manuscript. TF and RP prepared MSC populations and performed some experiments with flow cytometry. MP and HZ performed metabolic related experiments. DR helped with multiplex analysis and edited the manuscript. PH provided bone marrow aspirates. GM edited the manuscript. RC conceived and supervised the studies, analyzed and interpreted the data and wrote the manuscript. All the authors approved the manuscript.

## Glossary

**Table:**

AHR	Aryl Hydrocarbon Receptor
AUC	Area Under Curve
DMSO	Dimethyl Sulfoxide
EGF	Epidermal Growth Factor
FGF-basic	Fibroblast growth factor 2
G-CSF	Granulocyte-Colony Stimulating Factor
GM-CSF	granulocyte-macrophage colony-stimulating factor
HGF	Hepatocyte Growth Factor
IFNγ	Interferon gamma
MCP-1	Monocyte chemoattractant protein-1
MHC I	Major Histocompatibility Complex I
MHC II	Major Histocompatibility Complex II
MIG	Monokine induced by gamma
MIP-1α	Macrophage inflammatory protein-1 alpha
MIP-1β	Macrophage Inflammatory Protein-1 beta
MNCs	Mononuclear cells
MSCs	Mesenchymal Stem/Stromal cells
MTT	3-(4,5-dimethylthiazol-2-yl)-2,5-diphenyltetrazolium bromide
NLP	Negative Logarithmic P
r	correlation coefficient
TCDD	2,3,7,8-Tetrachlorodibenzo-p-dioxin
VEGF	Vascular Endothelial Growth Factor.
