# Supplementary material for: Effects of Atrazine exposure on human bone marrow-derived mesenchymal stromal cells assessed by combinatorial assay matrix
Source: Front Immunol. 2023 Jul 31;14:1214098. doi: 10.3389/fimmu.2023.1214098 (PMC10426140; doi:10.3389/fimmu.2023.1214098)
Supplement: Supplementary file 1 [file DataSheet_1.pdf]

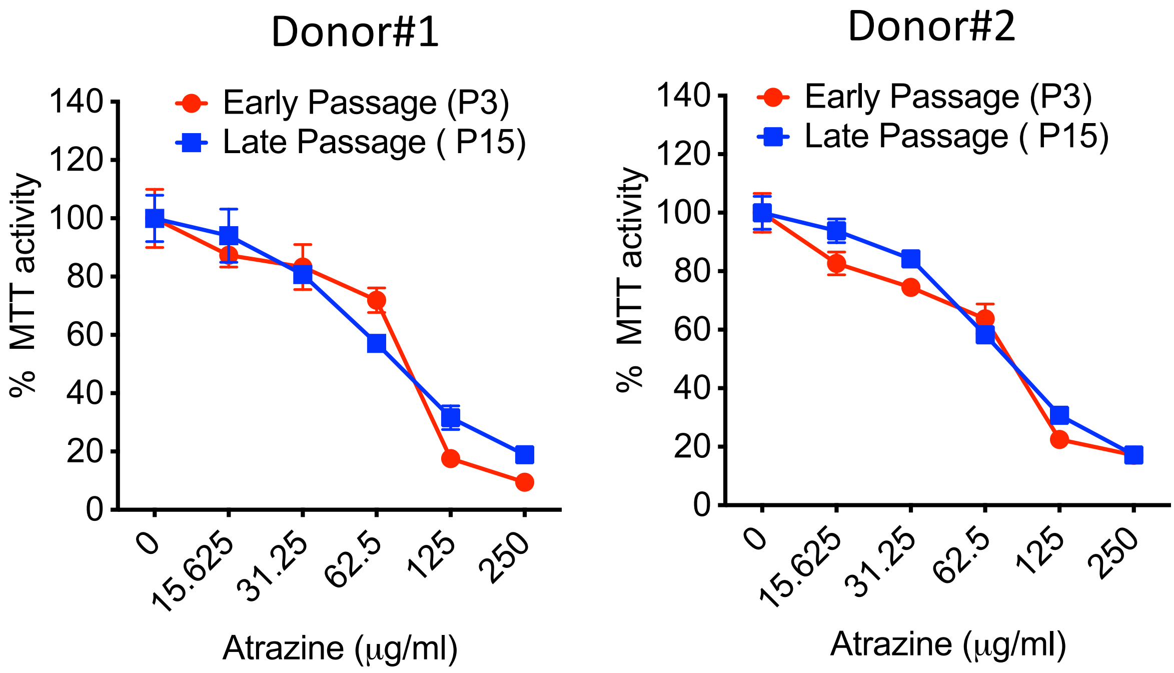

Figure S1. Effect of Passage difference on MSC sensitivity to Atrazine. Early or Late passage human MSCs derived from two independent donors were subjected to Atrazine exposure for seven days and MTT assay was performed. Dose dependent effect of Atrazine on % MTT activity is shown for each MSC donor.

Figure S1
