# Supplementary material for: Effects of Atrazine exposure on human bone marrow-derived mesenchymal stromal cells assessed by combinatorial assay matrix
Source: Front Immunol. 2023 Jul 31;14:1214098. doi: 10.3389/fimmu.2023.1214098 (PMC10426140; doi:10.3389/fimmu.2023.1214098)
Supplement: Supplementary file 2 [file DataSheet_2.pdf]

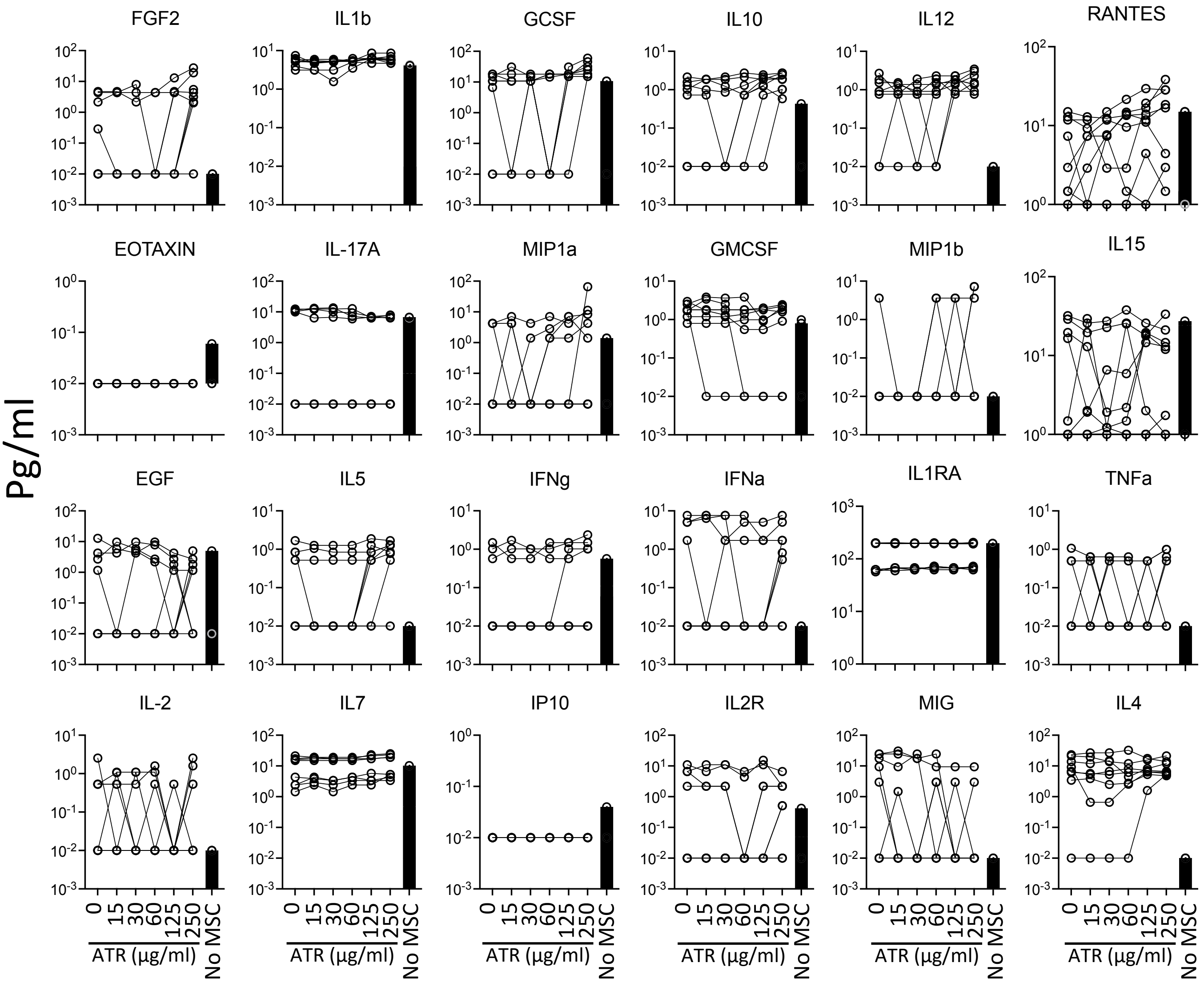

Figure S2. Atrazine does not induce de novo secretion cytokines and growth factors on MSCs. Human MSCs derived from eight independent donors were seeded in a 96 well plate and treated with the indicated concentrations of Atrazine (µg/ml). After seven days of culture, supernatants were collected and analyzed for 30-plex secretome using Luminex^{TM} xMAP (multi-analyte profiling) technology. 24 cytokines or growth factors that are not innately secreted by MSCs and are not upregulated upon Atrazine exposure are shown. No MSC (Media only) control is also shown. Please also refer to Figure 2.

Figure S2
