# Supplementary material for: Effects of Atrazine exposure on human bone marrow-derived mesenchymal stromal cells assessed by combinatorial assay matrix
Source: Front Immunol. 2023 Jul 31;14:1214098. doi: 10.3389/fimmu.2023.1214098 (PMC10426140; doi:10.3389/fimmu.2023.1214098)
Supplement: Supplementary file 3 [file DataSheet_3.pdf]

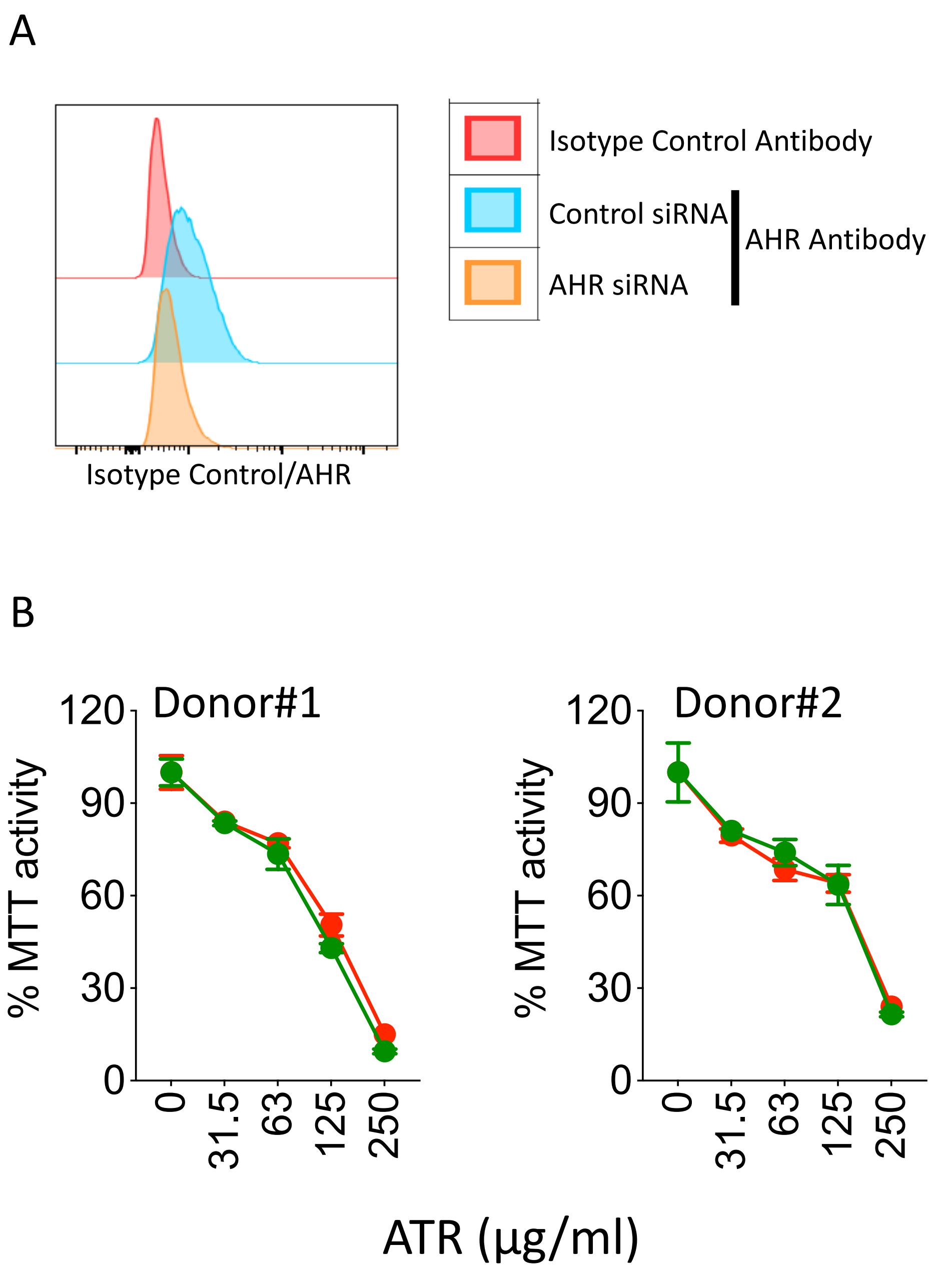

Figure S3. Role of AHR on MSC's metabolic activity. A. AHR Knock down efficiency is confirmed on control or AHR siRNA silenced human MSCs through flow cytometry. **B.** Control or AHR siRNA silenced human MSCs derived from two independent donors were subjected to Atrazine exposure for seven days and MTT assay was performed. Dose dependent effect of Atrazine on % MTT activity is shown for each MSC donor.

Figure S3
